# Supplementary material for: IF1 promotes oligomeric assemblies of sluggish ATP synthase and outlines the heterogeneity of the mitochondrial membrane potential
Source: Commun Biol. 2023 Aug 12;6:836. doi: 10.1038/s42003-023-05214-1 (PMC10423274; doi:10.1038/s42003-023-05214-1)
Supplement: Supplementary file 2 — Supplementary Information [file 42003_2023_5214_MOESM2_ESM.pdf]

Supplementary Information

IF1 promotes oligomeric assemblies of sluggish ATP synthase and outlines the heterogeneity of the mitochondrial membrane potential

Inés Romero-Carramiñana[1,2,3, #], Pau B. Esparza-Moltó[1,2,3, #], Sonia Domínguez-Zorita[1,2,3], Cristina Nuevo-Tapioles[1,2,3] and José M. Cuezva[1,2,3], *

Supplementary Figure 1: Jurkat cells contain a fraction of inactive ATP synthase bound to IF1 under basal conditions. (a) Proliferation rate of CRL and IF1-KO HCT116 cells (n=4). **(b)** Representative immunofluorescence illustrating the colocalization of IF1 (red) and β-F1-ATPase (green) in HCT116 cells. DAPI (blue) stained nuclei. **(c)** Representative blot of IF1 expression in extracts of Jurkat CRL cells and two different CRISPR single guide RNAs (sg) for *Atp5if1* gene (IF1-KO). β-actin is shown as loading control. Molecular weight markers are indicated to the right of the blots. **(d, e)** Representative histograms showing the oligomycin sensitive synthetic **(d)** and hydrolytic **(e)** activities of ATP synthase in digitonin-permeabilized **(d)** or isolated mitochondria **(e)** from CRL and IF1-KO Jurkat cells (n=4). 2 μM Oligomycin (OL) was used to inhibit ATP synthase activities. **(f)** Representative images of Proximity Ligation Assay (PLA) showing the interaction between β-F1-ATPase and IF1 (green dots) in CRL and IF1-KO Jurkat cells. DAPI (blue) stained nuclei. Histograms show the number of PLA signals per cell in CRL (n=3) and IF1-KO (n=3) Jurkat cells. **(g)** Representative profiles of oxygen consumption rates (OCR) of Jurkat CRL and IF1-KO cells (n=6) with glucose as respiratory substrate. The addition of oligomycin (OL), 2,4-dinitrophenol (DNP) and rotenone (R) plus antimycin A (A) is indicated. **(h)** Glycolytic activity measured by the rate of lactate production in CRL and IF1-KO Jurkat cells (n=5). **(d-h)** Experiments were carried out with sg1 IF1-KO cells. "n" indicates the number of independent experiments. The histograms and plots represent the mean and the error bars the ± SEM. $*p \leq 0.05$; $**p \leq 0.01$ when compared to CRL by Student's t test. Related to data on Figure 1 and 2.

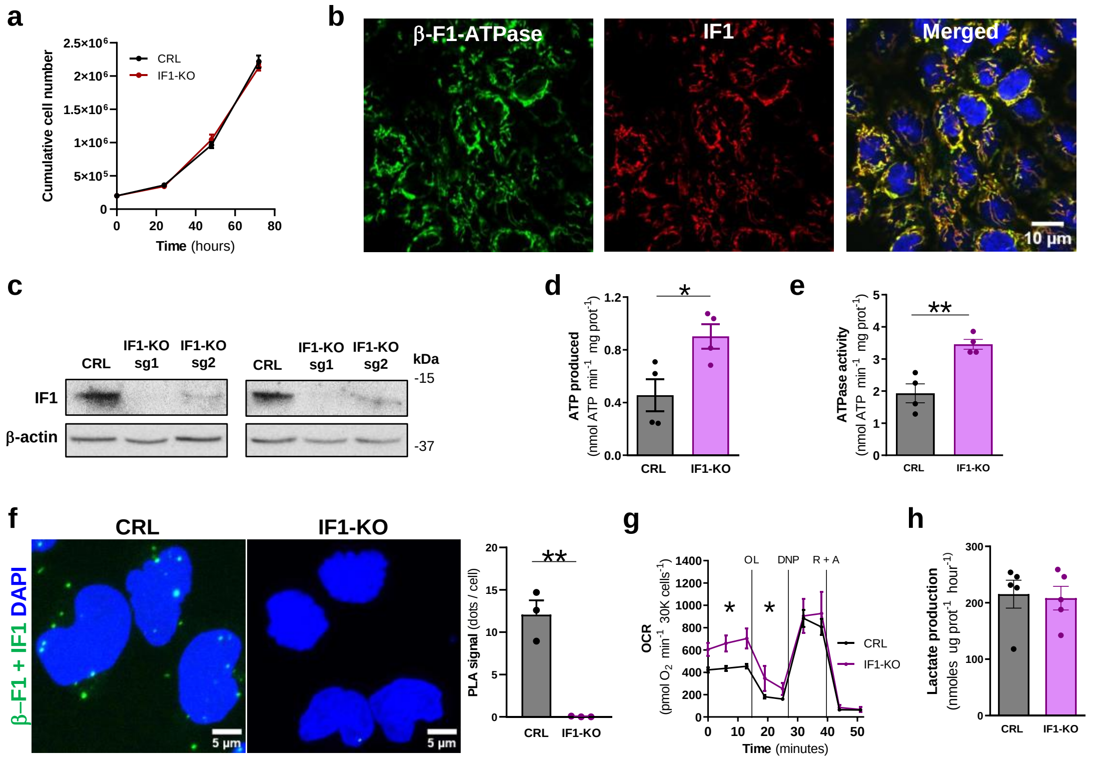

Supplementary Figure 2: The expression and activity of complexes of the respiratory chain are not affected in IF1-KO HCT116 cells. (a) Histograms show the enzymatic activity of mitochondrial complex I (n=3), II (n=2-3) and IV (n=3) of CRL and IF1-KO HCT116 cells. **(b)** Representative blots of the expression of subunits of mitochondrial complexes I (NDUFA9), II (SDHB), III (UQCRC2), IV (COX IV), V (β- and γ-F1-ATPase) and HSP60 in cell extracts of CRL and IF1-KO HCT116 cells. β-actin is shown as loading control. Histograms show the expression levels relative to β-actin (n=4). **(c)** Representative immunofluorescence illustrating the mitochondrial colocalization of NDUFS5 (green) and UQCRCII (red) in CRL and IF1-KO HCT116 cells. DAPI (blue) stained nuclei. Molecular weight markers are indicated to the right of the blots. **(d)** Violin plots showing the area and circularity (n=29-39) of mitochondria in electron micrographs of CRL and IF1-KO HCT116 cells. **(e)** Representative blots of the expression of OPA1, MIC60 and IF1 in cell extracts of CRL and IF1-KO HCT116 cells. β-actin is shown as loading control. Histograms show the expression levels relative to β-actin (n=4). Molecular weight markers are indicated to the right of the blots. **(f)** Representative immunofluorescence illustrating the mitochondrial colocalization of γ-F1-ATPase (γ-F1, green) and β-F1-ATPase (β-F1, red) in CRL and IF1-KO HCT116 cells. DAPI (blue) stained nuclei. **(g)** Representative images of PLA using γ-F1-ATPase as target (green dots) in Jurkat CRL and IF1-KO cells. DAPI (blue) stained nuclei. Histograms show the number of PLA signals per cell in CRL (n=3) and IF1-KO (n=3) Jurkat cells. "n" indicates the number of independent experiments. The histograms represent the mean and the error bars the ± SEM. *$p \leq 0.05$; ***$p \leq 0.001$ when compared to CRL by Student's t test. Related to data on Figure 2.

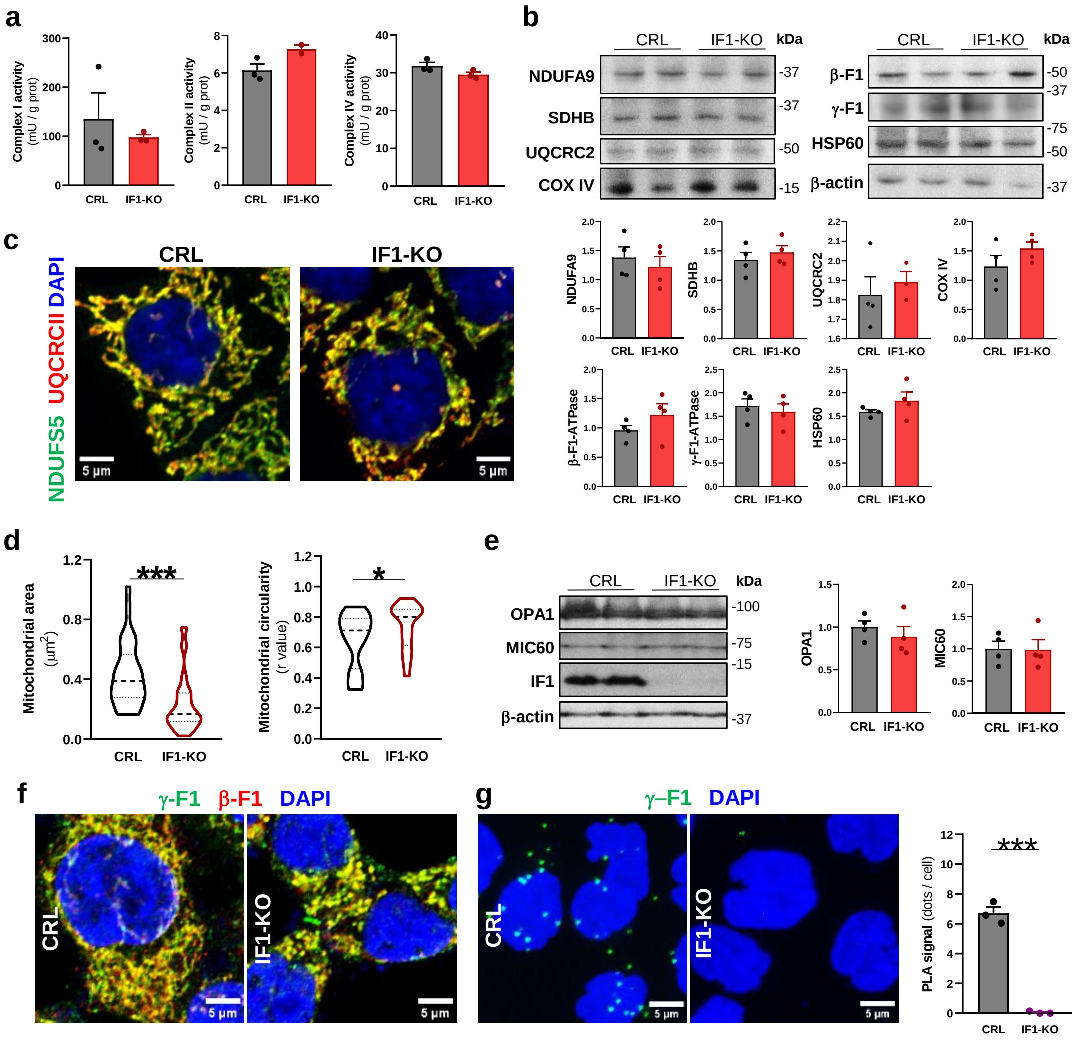
a
Complex I activity (mU / g prot)
Complex II activity (mU / g prot)
Complex IV activity (mU / g prot)
CRL
IF1-KO
b
CRL
IF1-KO
kDa
NDUFA9
SDHB
UQCRC2
COX IV
β-F1
γ-F1
HSP60
β-actin
β-F1-ATPase
γ-F1-ATPase
c
CRL
IF1-KO
NDUFS5 UQCRCII DAPI
5 µm
d
Mitochondrial area (μm^2)
Mitochondrial circularity (r value)
***
*
e
OPA1
MIC60
IF1
f
γ-F1 β-F1 DAPI
g
γ–F1 DAPI
PLA signal (dots / cell)

Supplementary Figure 3: Live cell imaging of the intramitochondrial distribution of IF1 and mtROS. (a) Representative immunofluorescences illustrating the colocalization between GFP signal (green) and mitochondria (MT-CO1; red) in IF1-GFP, IF1-S39E-GFP and pβ-GFP HCT116 cells. DAPI (blue) stained nuclei. Histograms represent the Pearson's correlation coefficient between GFP and MT-CO1 signal. The histograms represent the mean and the error bars the ± SEM **(b)** Representative high-resolution images of mitochondria from IF1-GFP and pβ-GFP HCT116 live cells stained with MitoSOX. Scale bars 2 µm. **(c)** Violin plots show the Manders' colocalization coefficients of MitoSOX signals and GFP fluorescence pixel by pixel of IF1-GFP and pβ-GFP images. Representative data of ten different fields from one experiment. Images were aligned and deconvoluted via Hyugens software and analyzed with the JACoP plugin of ImageJ. M1 represents the proportion of high MitoSOX signals that overlap with GFP signals and M2 the proportion of GFP signals that overlap with high MitoSOX signals. In both MitoSOX and GFP fluorescence, the threshold used only selected the pixels with maximal fluorescence intensity of GFP and MitoSOX. n.s., no significant. Related to data on Figure 3.

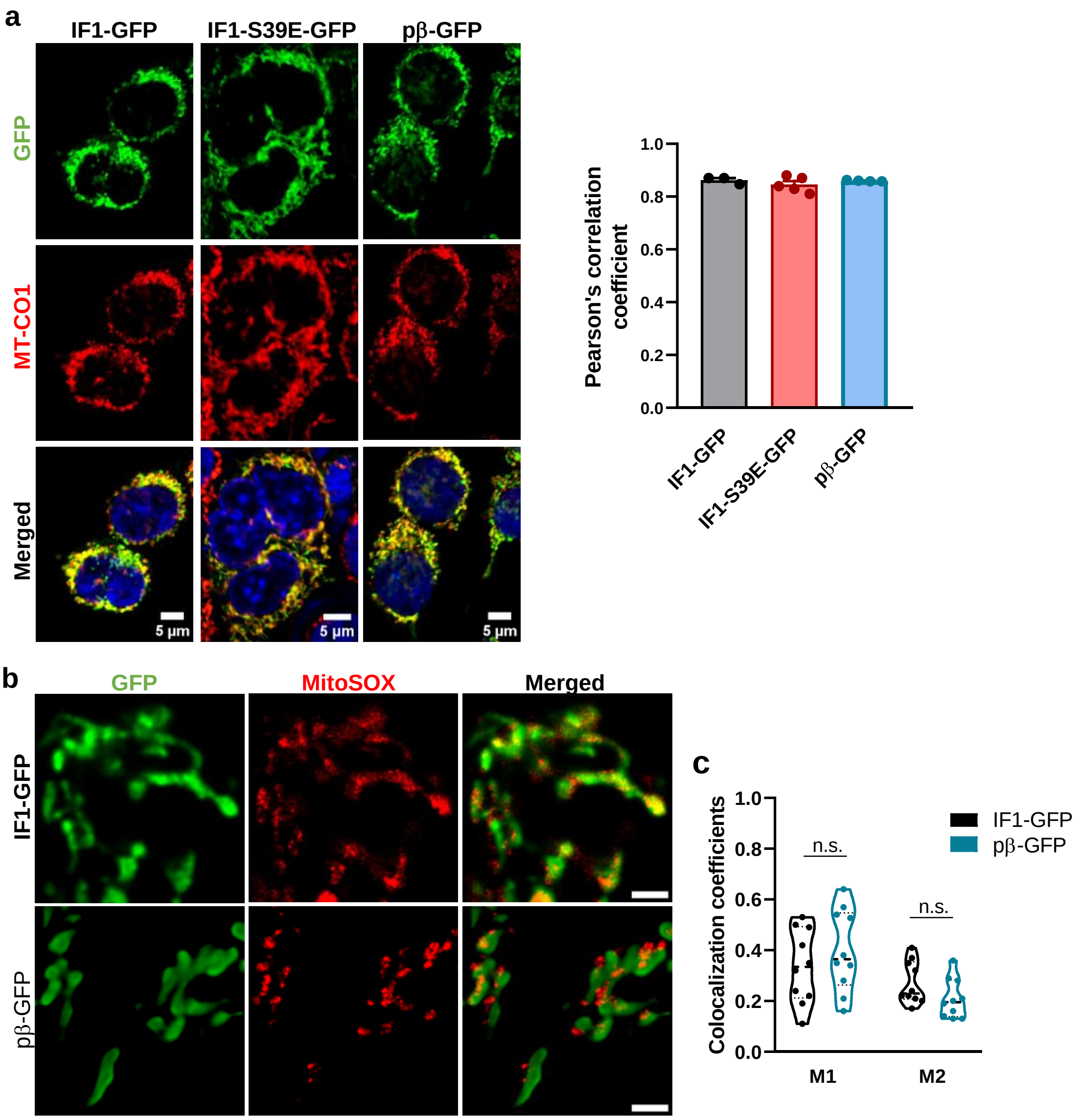

Supplementary Figure 4: Intramitochondrial distribution of ATP synthase and IF1 in mouse tissues. (a) Left, histograms show the mean and the error bars the ± SEM of the weight of CRL (n=8) and IF1-KO (n=5) male mice at 10 weeks of age. Right, representative image of CRL and IF1-KO mice. **(b)** Representative electron micrographs of mouse heart mitochondria showing the distribution of β-F1 particles (arrowheads) in cristae tips (left up), cristae sheets (left down), cristae junctions (right up) and IBM (right down). ATP synthase labeling of the sheet represents both enzyme at the sheet and at the rim of the sheet, because ATP synthase decorates cristae rims all along 45. Hence, the labeling at the sheet is an overestimation of enzyme at this location. Scale bars, 100 nm. **(c)** Stacked bars representing the distribution of β-F1 (n=729-925) and IF1 (n=153-225) gold particles in the indicated mitochondrial compartments in mouse kidney and heart mitochondria. IBM, inner boundary membrane. Related to data on Figures 4 and 5.

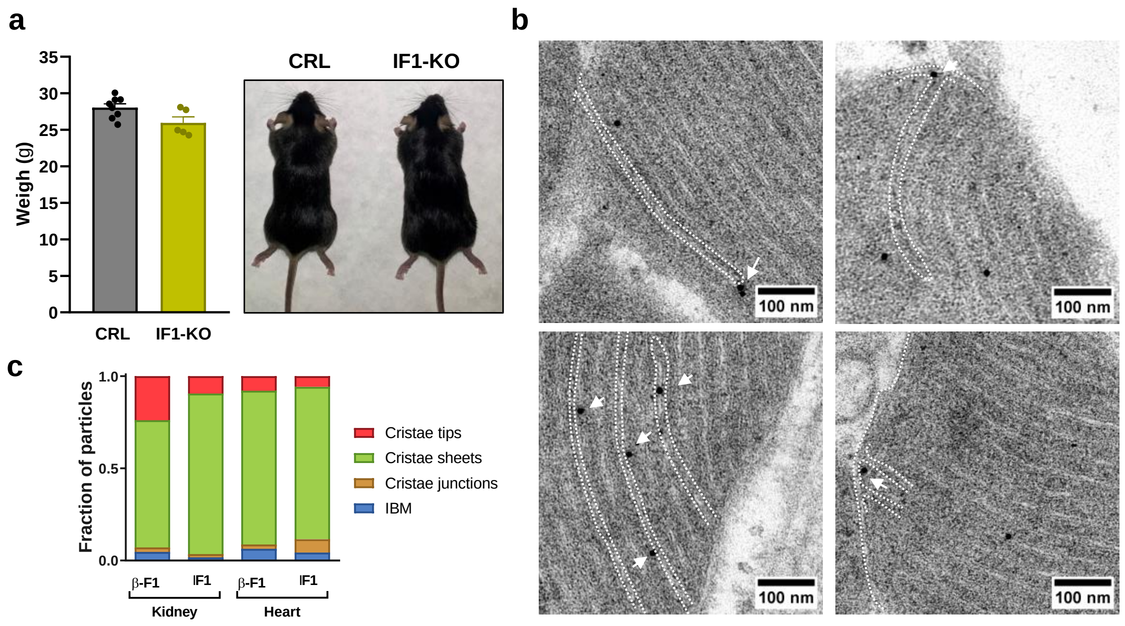

Supplementary Figure 5: Gating strategies for flow cytometry analysis. (a, b) Representative gating strategies to measure mitochondrial membrane potential with TMRM **(a)** and mitochondrial ROS production with MitoSOX **(b)** in CRL and IF1-KO HCT116 cells presented in Fig. 2c and 2d. First, cells are selected by size and complexity (1), doublets were excluded (2) and the TMRM **(a)** or MitoSOX **(b)** fluorescence (4) is obtained of DAPI unstained cells (3).

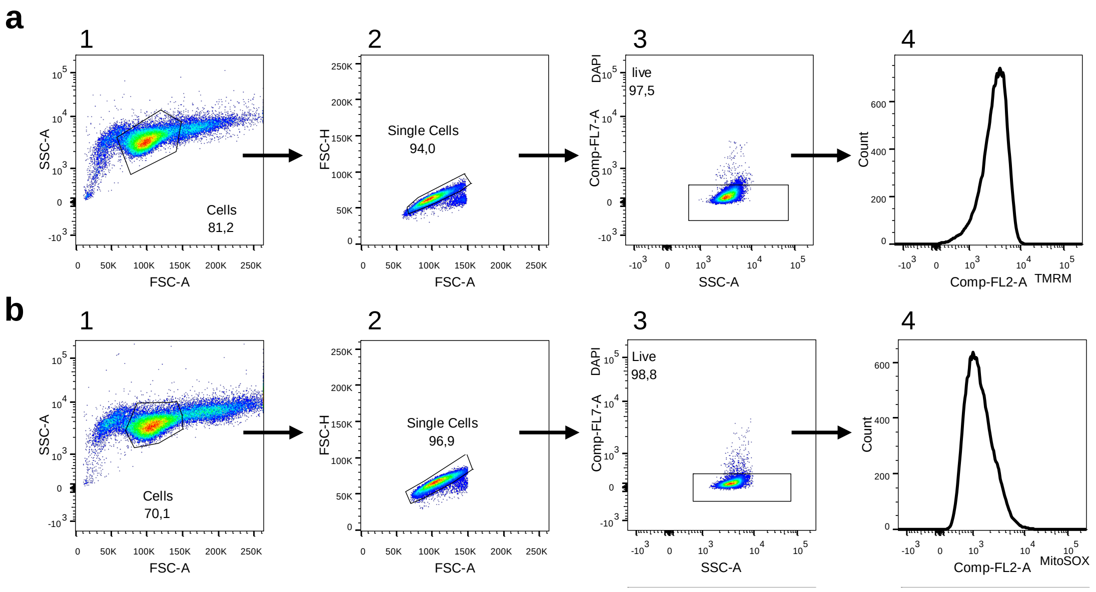

Supplementary Figure 6: Uncropped blots for Figure 1. Uncropped and unedited blots and gel of Figure 1 panel a (**a**), e (**b**) and g (**c**).

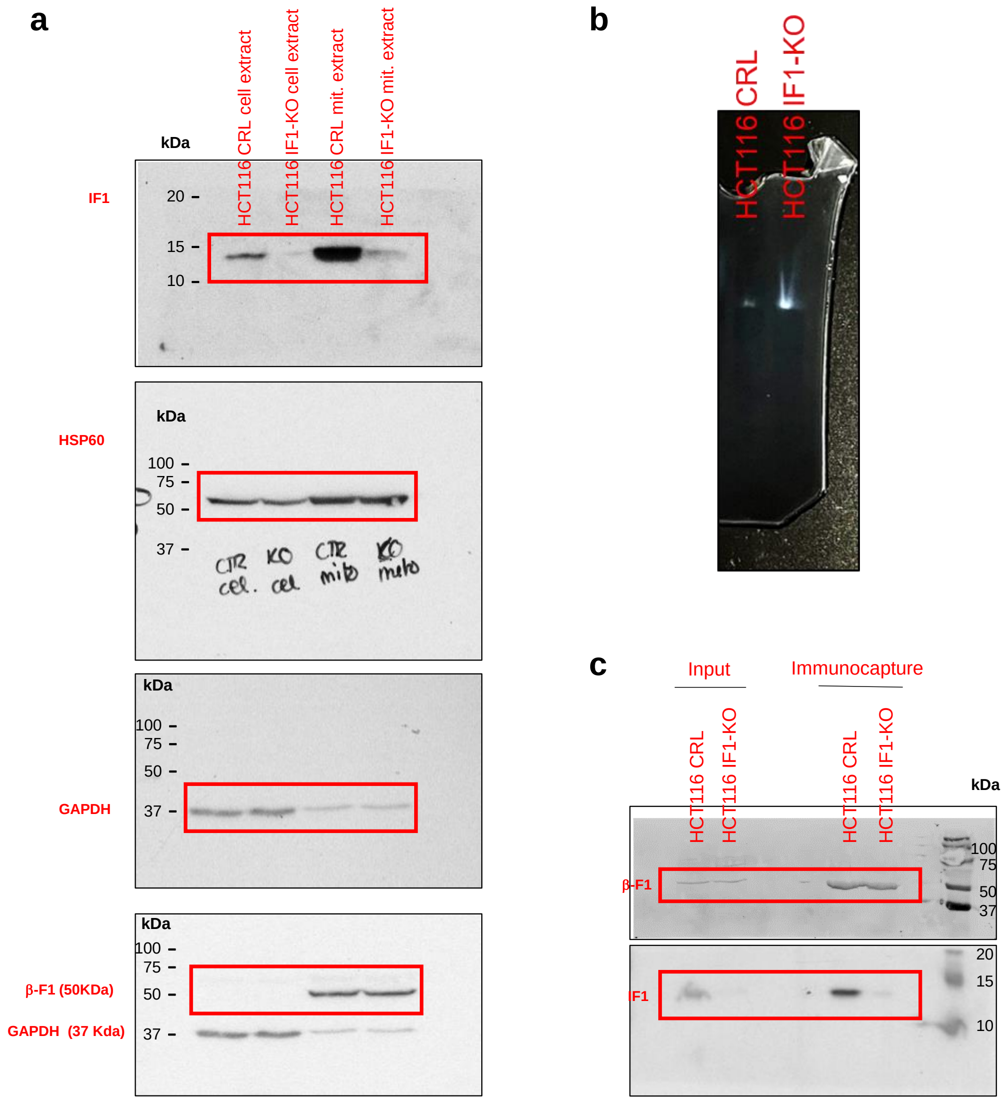

Supplementary Figure 7: Uncropped blots for Figure 2. Uncropped and unedited blots of Figure 2 panel e.

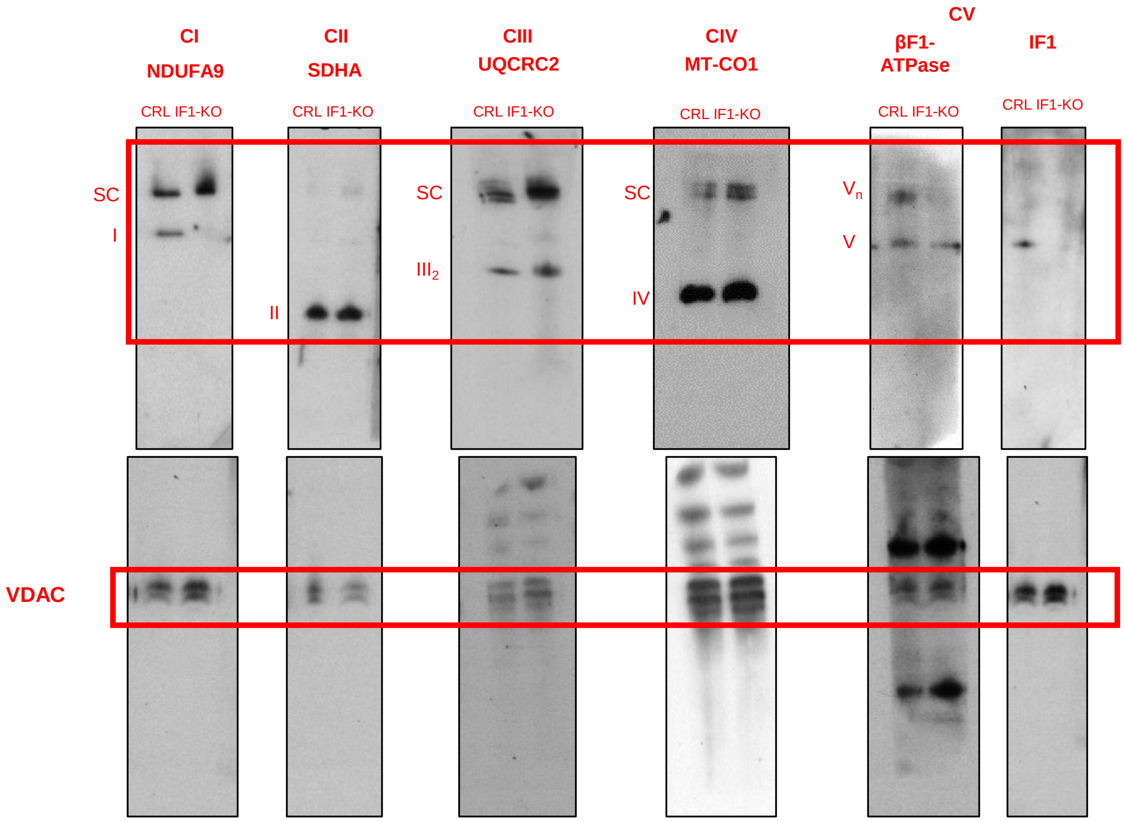

Supplementary Figure 8: Uncropped blots for Figure 3. Uncropped and unedited blots of Figure 3 panel a (**a**), b (**b**), c (**c**) and d (**d**).

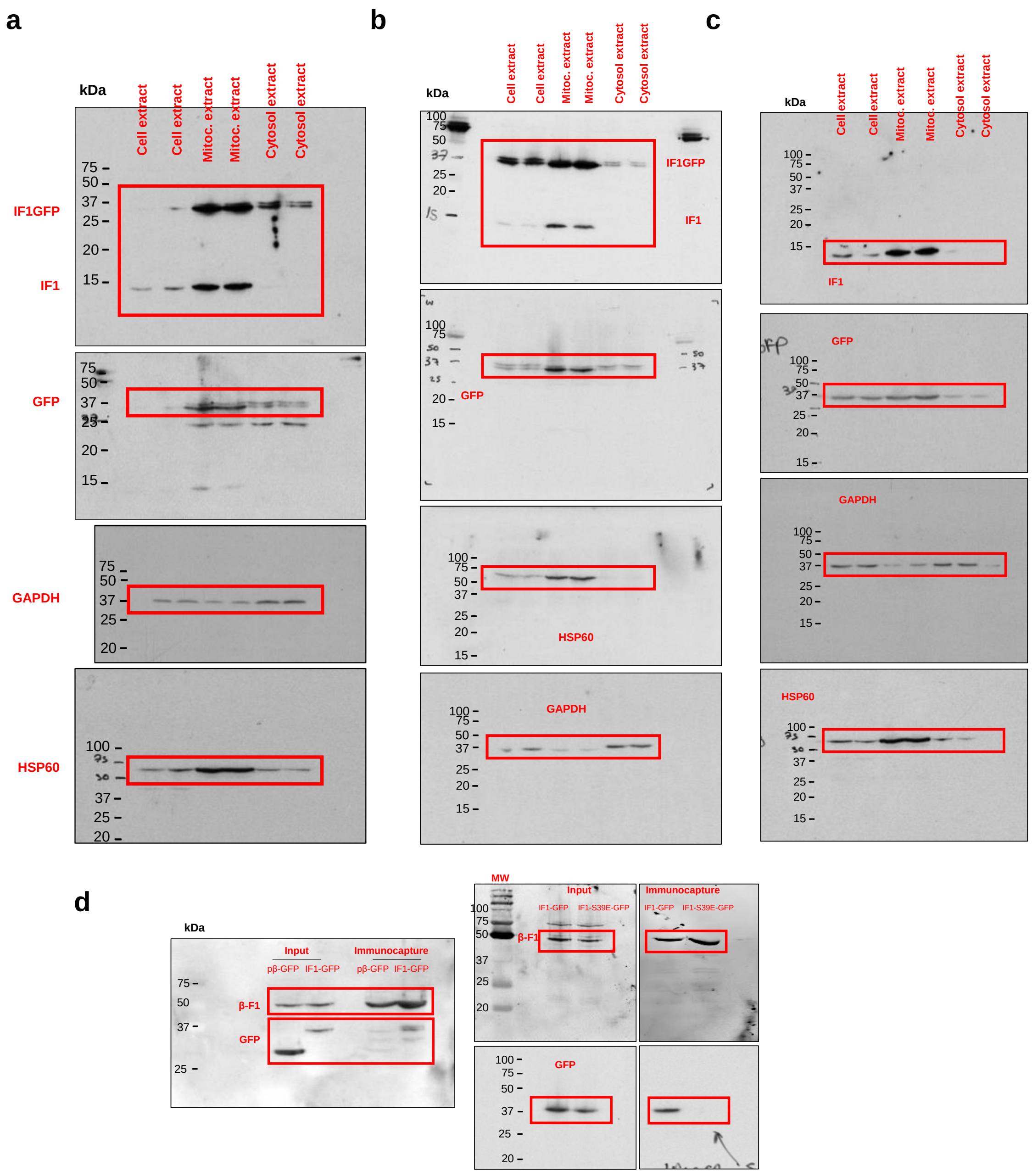

Supplementary Figure 9: Uncropped blots for Figure 4. Uncropped and unedited blots and gels of Figure 4 panel b (**a**), e (**b**), f (**c**), g (**d**) and h (**e**).

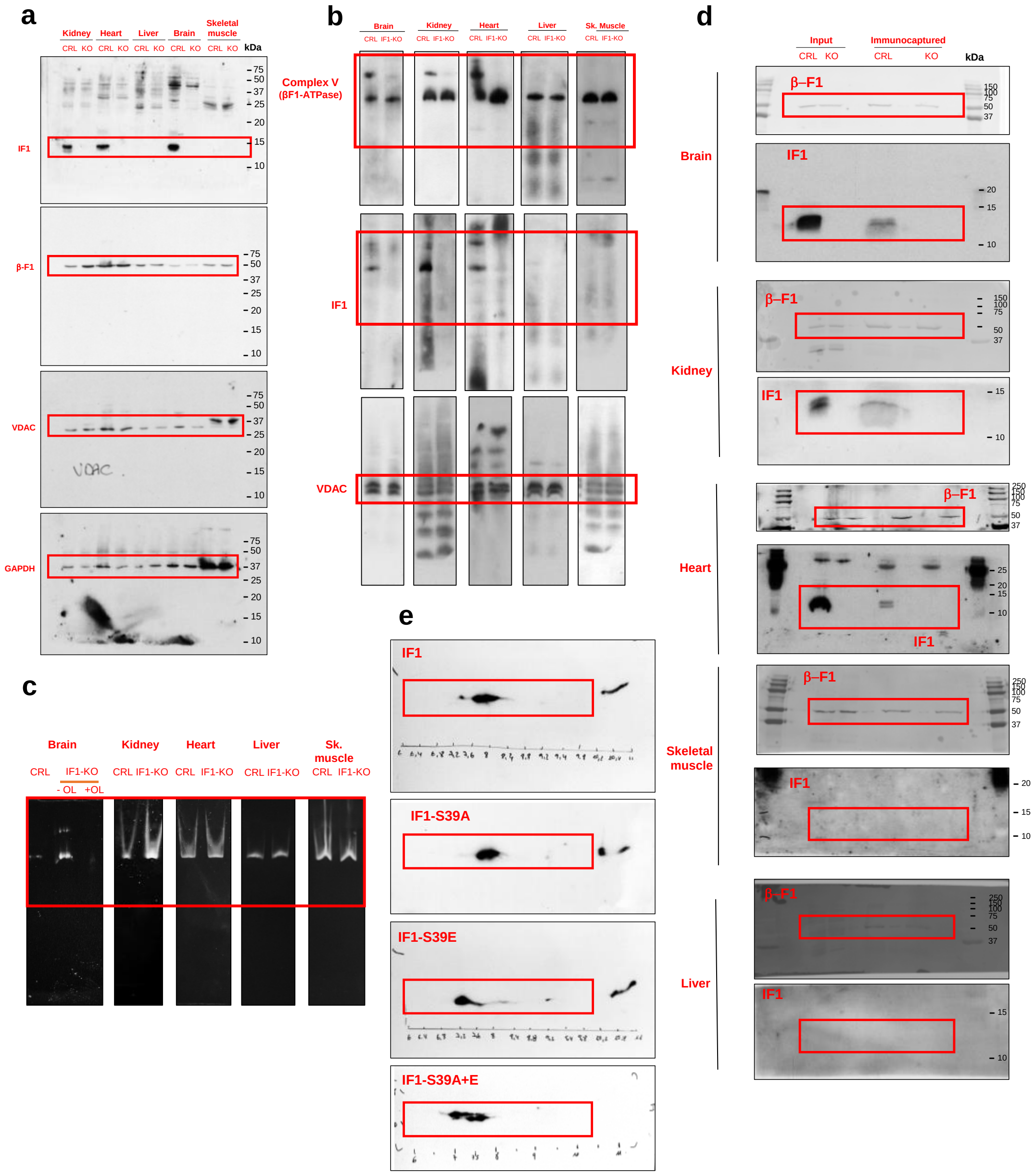

Supplementary Figure 10: Uncropped blots for Supplementary Figure 2. Uncropped and unedited blot of Supplementary Figure 1 panel c.

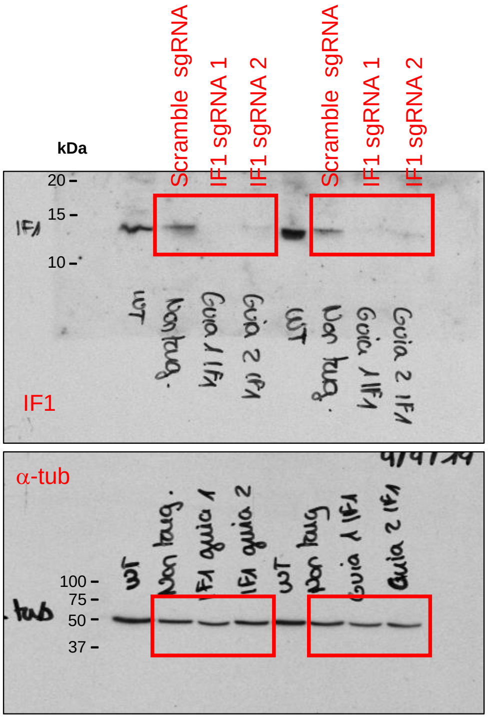

Supplementary Figure 11: Uncropped blots for Supplementary Figure 2. Uncropped and unedited blots of Supplementary Figure 2 panel b (**a**) and e (b).

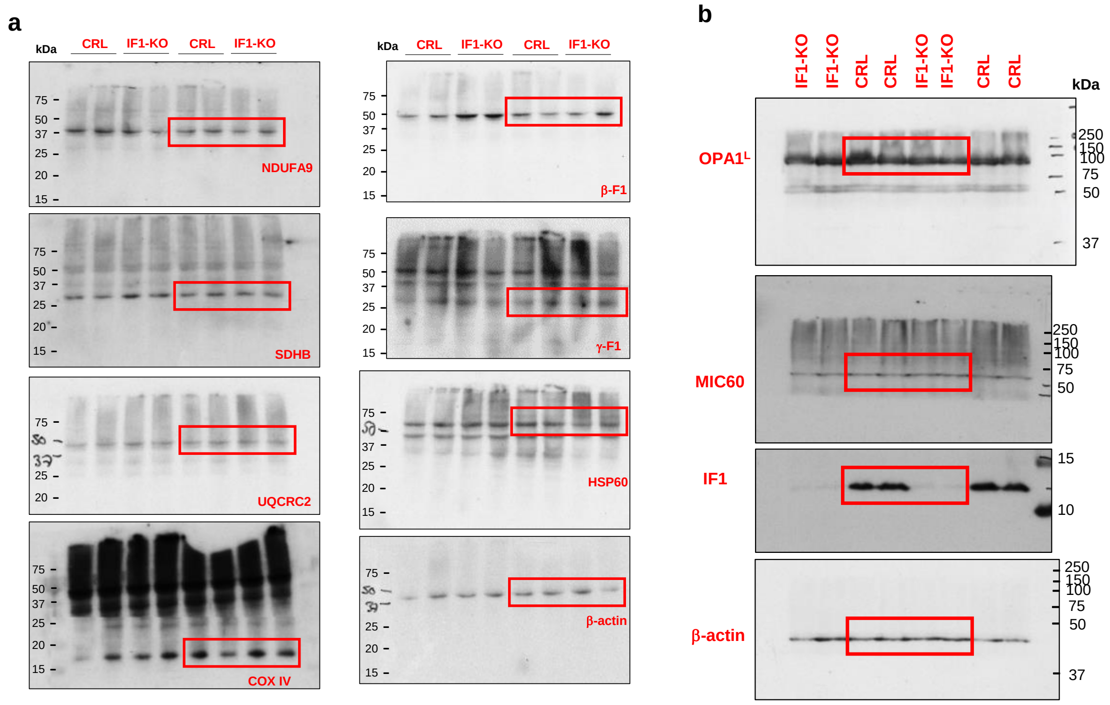

Supplementary Table 1. **Source of the specific antibodies used.** N/A; not apply.

Antibody	Usage	SOURCE	IDENTIFIER
Rabbit anti-mouseIF1	1:2000	José M. Cuezva (Esparza-Moltó et al., 2019)	N/A
Mouse anti-humanIF1 (clone 14/2)	1:1000	José M. Cuezva (Sánchez-Cenizo et al., 2010)	N/A
Rabbit anti-humanIF1	1:5000	This Paper	N/A
Mouse anti-β-F1 (clone 11/21-7A8)	1:1000	José M. Cuezva (Acebo et al., 2009)	N/A
Rabbit anti-β-F1	1:5000	José M. Cuezva (Cuezva et al., 2002)	N/A
Mouse anti-GAPDH (clone 273A-E5)	1:1000	José M. Cuezva (Acebo et al., 2009)	N/A
Mouse anti-HSP60 (clone 17/9-15 G1)	1:5000	José M. Cuezva (Acebo et al., 2009)	N/A
Mouse anti-SDHB (clone 21A11AE7)	1:1000	Invitrogen	Cat# 459230; RRID: AB_2532233
Mouse anti-COX IV (clone 20E8C12)	1:1000	Abcam	Cat# ab14744; RRID: AB_301443
Mouse anti-β-actin (clone AC-74)	1:10000	MilliporeSigma	Cat# A5316; RRID: AB_476743
Rabbit anti-VDAC1	1:500	Abcam	Cat# ab15895; RRID: AB_2214787
Mouse anti-NADHs9 (NDUFA9, clone 15/22-5)	1:1000	José M. Cuezva (Santacatterina et al., 2015)	N/A
Mouse anti-SDHA (clone 2E3GC12FB2AE2)	1:1000	Abcam	Cat# ab14715; RRID: AB_301433
Mouse anti-MT-CO1 (clone 1D6E1A8)	1:1000	Invitrogen	Cat# 459600; RRID: AB_2532240
Rabbit anti-GFP	1:3000	ChromoTek	Cat# PABG1; RRID: AB_2749857
Mouse anti-UQCRC2 (CORE II, clone 13G12AF12BB11)	1:1000	Abcam	Cat# ab14745; RRID: AB_2213640
Rabbit anti-NDUFS5	1:1000	Proteintech	Cat#15224-1-AP RRID: AB_2149021
Mouse anti-γ-F1	1:500	José M. Cuezva (Willers et al., 2012)	N/A
Peroxidase-conjugated rabbit anti-mouse IgGs	1:5000	Nordic Immunology	Cat# RAM/IgG(H+L)/PO
Peroxidase-conjugated goat anti-rabbit IgGs	1:5000	Nordic Immunology	Cat# GAR/IgG(H+L)/PO
Alexa 488-conjugated donkey anti-mouse IgGs	1:2000	Thermo Fisher	Cat# R37114; RRID: AB_2556542
Alexa 555-conjugated donkey anti-rabbit IgGs	1:2000	Thermo Fisher	Cat# A-31572; RRID: AB_162543
Alexa 555-conjugated donkey anti-mouse IgGs	1:2000	Thermo Fisher	Cat# A-31570; RRID: AB_2536180
