# Supplementary material for: IF1 promotes oligomeric assemblies of sluggish ATP synthase and outlines the heterogeneity of the mitochondrial membrane potential
Source: Commun Biol. 2023 Aug 12;6:836. doi: 10.1038/s42003-023-05214-1 (PMC10423274; doi:10.1038/s42003-023-05214-1)
Supplement: Supplementary file 5 — Reporting Summary [file 42003_2023_5214_MOESM5_ESM.pdf]

nature portfolio

Corresponding author(s): José M. Cuezva
Last updated by author(s): Jul 31, 2023

Reporting Summary

Nature Portfolio wishes to improve the reproducibility of the work that we publish. This form provides structure for consistency and transparency in reporting. For further information on Nature Portfolio policies, see our Editorial Policies and the Editorial Policy Checklist.

Statistics

For all statistical analyses, confirm that the following items are present in the figure legend, table legend, main text, or Methods section.

n/a	Confirmed	
☐	☒	The exact sample size (*n*) for each experimental group/condition, given as a discrete number and unit of measurement
☐	☒	A statement on whether measurements were taken from distinct samples or whether the same sample was measured repeatedly
☐	☒	The statistical test(s) used AND whether they are one- or two-sided<br>*Only common tests should be described solely by name; describe more complex techniques in the Methods section.*
☐	☒	A description of all covariates tested
☒	☐	A description of any assumptions or corrections, such as tests of normality and adjustment for multiple comparisons
☐	☒	A full description of the statistical parameters including central tendency (e.g. means) or other basic estimates (e.g. regression coefficient) AND variation (e.g. standard deviation) or associated estimates of uncertainty (e.g. confidence intervals)
☐	☒	For null hypothesis testing, the test statistic (e.g. *F*, *t*, *r*) with confidence intervals, effect sizes, degrees of freedom and *P* value noted<br>*Give P values as exact values whenever suitable.*
☒	☐	For Bayesian analysis, information on the choice of priors and Markov chain Monte Carlo settings
☒	☐	For hierarchical and complex designs, identification of the appropriate level for tests and full reporting of outcomes
☐	☒	Estimates of effect sizes (e.g. Cohen's *d*, Pearson's *r*), indicating how they were calculated

Our web collection on statistics for biologists contains articles on many of the points above.

Software and code

Policy information about availability of computer code

Data collection	We used Bio-Rad GS-900 for collecting WB data; BD FACSCanto II to collect flow cytometry data; Nikon A1R+ and Leica SP8 LSM fitted with STED module to collect confocal and STED microscopy images; JEM-1400Flash transmission electron microscope (JEOL) coupled to a CMOS OneView (4Kx4K) camera to collect electronic microscopy images; eFlux Analyzers XF96 and XF24 to obtain the mitochondrial oxygen consumption parameters and Omega FLUOstar control reader to mesure the different enzymatic activities.
Data analysis	Images analysis were performed using ImageJ software v1.5. Flow cytometry was analyzed using FlowJO software v10. Seahorse experiments were analyzed using Seahorse Wave v2.4. All the statistical analyses were performed by GraphPad Prism v8.

For manuscripts utilizing custom algorithms or software that are central to the research but not yet described in published literature, software must be made available to editors and reviewers. We strongly encourage code deposition in a community repository (e.g. GitHub). See the Nature Portfolio guidelines for submitting code & software for further information.

Data

Policy information about availability of data

All manuscripts must include a data availability statement. This statement should provide the following information, where applicable:

- Accession codes, unique identifiers, or web links for publicly available datasets
- A description of any restrictions on data availability
- For clinical datasets or third party data, please ensure that the statement adheres to our policy

Source data is provided with this paper as a file named Supplementary Data 1

Human research participants

Policy information about studies involving human research participants and Sex and Gender in Research.

Reporting on sex and gender	non applicable
Population characteristics	N/a
Recruitment	N/a
Ethics oversight	N/a

Note that full information on the approval of the study protocol must also be provided in the manuscript.

Field-specific reporting

Please select the one below that is the best fit for your research. If you are not sure, read the appropriate sections before making your selection.

☒ Life sciences ☐ Behavioural & social sciences ☐ Ecological, evolutionary & environmental sciences

For a reference copy of the document with all sections, see nature.com/documents/nr-reporting-summary-flat.pdf

Life sciences study design

All studies must disclose on these points even when the disclosure is negative.

Sample size	Required sample size were determined based on experience from previous experiments performed in our laboratory which reached statistically relevant results as shown in Nuevo-Tapioles et al., Nat. Commun. 2020; Esparza-Moltó FASEB J. 2019 and Esparza-Moltó PLoS Biol. 2021.
Data exclusions	No data were excluded.
Replication	All the experiments were repeated for at least 3 times except for Complex II activity (Supplementary Figure2 a) that was repeated only twice. All the attempts were successful.
Randomization	Randomization was not relevant for experiments.
Blinding	Investigators were not blinded during data collection or analysis because the sample names contained information to allow the identification.

Reporting for specific materials, systems and methods

We require information from authors about some types of materials, experimental systems and methods used in many studies. Here, indicate whether each material, system or method listed is relevant to your study. If you are not sure if a list item applies to your research, read the appropriate section before selecting a response.

Materials & experimental systems

n/a	Involved in the study
☐	☒ Antibodies
☐	☒ Eukaryotic cell lines
☒	☐ Palaeontology and archaeology
☐	☒ Animals and other organisms
☒	☐ Clinical data
☒	☐ Dual use research of concern

Methods

n/a	Involved in the study
☒	☐ ChIP-seq
☐	☒ Flow cytometry
☒	☐ MRI-based neuroimaging

Antibodies

Antibodies used

The primary antibodies used were rabbit anti-mouseIF1 (generated and provided by Cuezva's lab), mouse anti-humanIF1 (clone 14/2, generated and provided by Cuezva's lab), rabbit anti-humanIF1 (generated and provided by Cuezva's lab), mouse anti-beta-F1 (clone 11/21-7A8, generated and provided by Cuezva's lab), rabbit anti-beta-F1 (generated and provided by Cuezva's lab), mouse anti-GAPDH (clone 273A-E5, generated and provided by Cuezva's lab), mouse anti-HSP60 (clone 17/9-15 G1 generated and provided by Cuezva's lab), mouse anti-SDHB (clone 21A11AE7, Invitrogen, Cat# 459230) mouse anti-COX IV (clone 20E8C12, Abcam, Cat# ab14744) mouse anti-a-actin (clone AC-74, MilliporeSigma, Cat# A5316), rabbit anti-VDAC1 (Abcam, Cat# ab15895), mouse anti-NADHs9 (NDUFA9, clone 15/22-5, generated and provided by Cuezva's lab), mouse anti-SDHA (clone 2E3GC12FB2AE2, Abcam, Cat# ab14715), mouse anti-MT-CO1 (clone 1D6E1A8, Invitrogen, Cat# 459600), rabbit anti-GFP ChromoTek, Cat# PABG1), mouse anti-UQCRC2 (clone 13G12AF12BB11, Abcam, Cat# ab14745), rabbit anti-NDUFS5 (15224-1-AP) and mouse anti-gamma-F1 (generated and provided by Cuezva's lab).

Validation

Rabbit anti-mouseIF1 was produced in the laboratory and validated in Esparza-Moltó et al., FASEB J 2019. Mouse anti-humanIF1 (clone 14/2) was produced in the laboratory and validated in Sánchez-Cenizo et al., J. Biol. Chem. 2010. Rabbit anti-humanIF1 was produced in the laboratory and validated in this study. Rabbit anti-beta-F1 was produced in the laboratory and validated in Cuezva et al., Cancer Res. 2002). Mouse anti-GAPDH (clone 273A-E5), mouse anti-HSP60 (clone 17/9-15 G1) and mouse anti-beta-F1 (clone 11/21-7A8) were produced in the laboratory and validated in Acebo et al., Transl. Oncol. 2009). Mouse anti-NADHs9 (NDUFA9, clone 15/22-5) was produced in the laboratory and validated in Santacatterina et al., J. Trans. Med. 2015. Mouse anti-gamma-F1 was produced in the laboratory and validated in Willers et al., Biochim. Biophys Acta-Bioenergetics 2012.
The other antibodies are commercially available and validated in the literature as cited on the manufacturer's website, as well as by the datasheet they provide:
Mouse anti-SDHB (clone 21A11AE7): https://www.thermofisher.com/antibody/product/SDHB-Antibody-clone-21A11AE7-Monoclonal/459230
Mouse anti-COX IV (clone 20E8C12): https://www.abcam.com/cox-iv-antibody-20e8c12-ab14744.html
Mouse anti-b-actin (clone AC-74): https://www.sigmaaldrich.com/ES/es/product/sigma/a5316
Rabbit anti-VDAC1: https://www.abcam.com/vdac1porin-antibody-mitochondrial-loading-control-ab15895.html
Mouse anti-SDHA (clone 2E3GC12FB2AE2): https://www.abcam.com/sdha-antibody-2e3gc12fb2ae2-ab14715.html
Mouse anti-MT-CO1 (clone 1D6E1A8): https://www.thermofisher.com/antibody/product/MTCO1-Antibody-clone-1D6E1A8-Monoclonal/459600
Rabbit anti-GFP ChromoTek: https://www.ptglab.com/products/GFP-antibody-rabbit-polyclonal-PABG1.htm
Mouse anti-UQCRC2 (clone 13G12AF12BB11): https://www.abcam.com/uqcrc2-antibody-13g12af12bb11-ab14745.html
Rabbit anti-NDUFS5: https://www.fishersci.es/shop/products/ndufs5-rabbit-anti-human-mouse-rat-polyclonal-proteintech-2/16884314

Eukaryotic cell lines

Policy information about cell lines and Sex and Gender in Research

Cell line source(s)

HCT116, Jurkat and NRK cell lines were obtained from ATCC (Manassas, VA, USA). The different HCT116 and Jurkat KO, GFP and IF1-GFP developed cell lines were developed from the original ATCC cells.

Authentication

Human cell lines were authenticated by STR-(microsatellite) genotyping by the Genomic Unit at Instituto de Investigaciones Biomédicas (CSIC-UAM).
Animal cell lines were not authenticated.

Mycoplasma contamination

All cell lines were tested as negative for mycoplasma contamination.

Commonly misidentified lines
(See ICLAC register)

There are no misidentified cell lines in this study.

Animals and other research organisms

Policy information about studies involving animals; ARRIVE guidelines recommended for reporting animal research, and Sex and Gender in Research

Laboratory animals

10 weeks old wild-type C57BL/6J mice and IF1-KO mice maintained in a C57BL/6J background were used in this study.
3 months old New Zealand White rabbits were used for rabbit anti-humanIF1 antibodies production.

Wild animals	There are no wild animals used in this study.
Reporting on sex	Experiments were performed with male and female mice.
Field-collected samples	There are no field-collected samples used in this study.
Ethics oversight	The Ethics Committee of Animal Experimentation (CSIC-UAM, CM PROEX 233/19) and the Institutional Review Board of UAM (CEI-101-1891-A325) approved the project.

Note that full information on the approval of the study protocol must also be provided in the manuscript.

Flow Cytometry

Plots

Confirm that:

☒ The axis labels state the marker and fluorochrome used (e.g. CD4-FITC).

☒ The axis scales are clearly visible. Include numbers along axes only for bottom left plot of group (a 'group' is an analysis of identical markers).

☒ All plots are contour plots with outliers or pseudocolor plots.

☒ A numerical value for number of cells or percentage (with statistics) is provided.

Methodology

Sample preparation	For the determination of the mitochondrial membrane potential, HCT116 CRL and IF1-KO cells were incubated with 50 nM TMRM in FACS buffer (1mM EDTA, 2% FBS in PBS) during 20 minutes at 37 ºC in constant shaking. Cells were washed twice in FACS buffer and resuspended in FACS buffer containing 0.5 µg/mL DAPI before analysis. For collapsing the mitochondrial membrane potential, 3 minutes before analysis TMRM stained HCT116 CRL and IF1-KO cells were incubated with 1µM FCCP. For multidrug channels inhibition, 1.6 µM cyclosporin H was added in the culture medium of CRL and IF1-KO cells 24 hours before assessing the $\Delta\Psi m$. For the determination of the mitochondrial ROS production, HCT116 CRL and IF1-KO cells were incubated with 2.5 µM MitoSOX in FACS buffer during 20 minutes at 37 ºC in constant shaking. Cells were washed twice in FACS buffer and resuspended in FACS buffer containing 0.5 µg/mL DAPI before analysis. Specificity of MitoSOX staining was assessed by the addition of 5 µM antimycin during MitoSOX incubation in HCT116 CRL and IF1-KO cells. For ATP synthase inhibition, 2 µM oligomycin was added during the TMRM or MitoSOX probe staining.
Instrument	The instrument used for data collection was a FACSCanto II High Throughput Sampler Option.
Software	Software data collection was performed with BD FACSDiva 6.6.2 software. Data analysis was done using FlowJO v10 cytometry software.
Cell population abundance	In all cases, at least 10,000 live events were recorded to measure mitochondrial membrane potential and ROS production.
Gating strategy	Cells were selected using the FSC/SSC gating. Single cells were selected using the FSC-Height and Area gating. Live cells were selected as DAPI negative cells. Mean fluorescence intensity of TMRM or MitoSOX probes was measured on DAPI negative cells.

☒ Tick this box to confirm that a figure exemplifying the gating strategy is provided in the Supplementary Information.
